# Patient-derived xenografts or organoids in the discovery of traditional and self-assembled drug for tumor immunotherapy

**DOI:** 10.3389/fonc.2023.1122322

**Published:** 2023-04-04

**Authors:** Wei Zhang, Xiaoqiang Zheng

**Affiliations:** ^1^ Department of Talent Highland, The First Affiliated Hospital of Xi’an Jiaotong University, Xi’an, China; ^2^ Department of Medical Oncology, The First Affiliated Hospital of Xi’an Jiaotong University, Xi’an, China; ^3^ Institute for Stem Cell & Regenerative Medicine, The Second Affiliated Hospital of Xi’an Jiaotong University, Xi’an, China

**Keywords:** drug discovery, PDO, PDX, tumor immunotherapy, tumor microenvironment

## Abstract

In addition to the rapid development of immune checkpoint inhibitors, there has also been a surge in the development of self-assembly immunotherapy drugs. Based on the immune target, traditional tumor immunotherapy drugs are classified into five categories, namely immune checkpoint inhibitors, direct immune modulators, adoptive cell therapy, oncolytic viruses, and cancer vaccines. Additionally, the emergence of self-assembled drugs with improved precision and environmental sensitivity offers a promising innovation approach to tumor immunotherapy. Despite rapid advances in tumor immunotherapy drug development, all candidate drugs require preclinical evaluation for safety and efficacy, and conventional evaluations are primarily conducted using two-dimensional cell lines and animal models, an approach that may be unsuitable for immunotherapy drugs. The patient-derived xenograft and organoids models, however, maintain the heterogeneity and immunity of the pathological tumor heterogeneity.

## 1 Introduction

Clinical sample sequencing and experiments using animal models have demonstrated that the molecular mechanism of tumorigenesis is due to gene mutations induced by oncogene and anti-oncogene. However, oncogene mutation is not the only factor that eventually causes the development of cancer (1–3). Several preclinical and clinical studies have reported that multiple factors exist between the occurrence of oncogene mutations in cells and the tumors *in situ*, such as the tumor microenvironment (TME) (4–6).TME as a concept was proposed by Ioannides in 1993 (7). Currently, TME is regarded as the presentation of non-tumor cells and their components in tumors, including the protein molecules produced and released by them(8). Furthermore, the metabolic disorders of TME cells result in the consumption of nutrients, acidification of environmental pH, hypoxia, and the production of regulatory metabolites, thus influencing the immune response to tumors as well as the overexpression of immune checkpoint molecules and tumor metastasis (9–11). The abortive phenomenon of various tumor therapy drugs in previous preclinical and clinical trials has been explained by the discovery of TME.

The concept of immunotherapy was first introduced by William Coley in the 1890s(12). Later, Honjo discovered that programmed death receptor 1 (PD-1) is an inducible gene on activated T lymphocytes, which led to the discovery of cancer immunotherapy through blocking PD-1 (13). Meanwhile, a protein on the molecular surface of immune cells called cytotoxic T lymphocyte-associated antigen 4 (CTLA-4) was discovered by James P. Allison to act as a “molecular brake” that prevents the immune system from responding. Inhibition of CTLA-4 can make T cells proliferate and attack tumor cells (14). The Nobel Prize in Physiology or Medicine was awarded to them in recognition of their contribution to tumor immunotherapy in 2018. Currently, tumor immunotherapy drugs can be mainly divided into five categories: antibody drugs such as immune checkpoint inhibitors (ICIs) (15), direct immune modulators (16–19), chimeric antigen receptor (CAR) -T cells(20), oncolytic viruses (OVs) (21) and cancer vaccines (22).Despite this progress, contributing to off-target toxicity, tissue heterogeneity, poor immunogenicity and tumor infiltration, the clinical use of tumor immunotherapy remains limited to a small subset of cancers. The development of self-assembly nanotechnology provides an opportunity for enhancing the effectiveness and reducing the toxicity of traditional drugs, and a series of nanomaterials were used in the preclinical study of cancer (23). This technology assembles molecules with different functions into highly ordered nanosystems with non-covalent bonds, which is a strategy for building powerful drugs (24).

In recent years, the development of experimental models to accurately replicate the pathophysiology of tumors has become one of the main challenges in the development of new drugs. Researchers emphasize patient-derived tumor xenografts (PDXs) (25)and patient-derived organoids (PDOs) (26) as potential solutions to these problems. PDX preserves the histological structure, degree of differentiation, morphological features, and molecular characteristics of most primary tumors and can better mimic their response to treatment. PDO models and three-dimensional (3D) culture can reproduce TME and biological behavior of tumor cells *in vitro* by reconstructing 3D communication networks of cell-cell and cell-extracellular matrix (ECM) interactions (16–19). Drug research and development have benefited greatly from the PDX and PDO models (27).

In this review, we discuss the latest advances of the technology in PDX and PDO models for tumor immunotherapy research. We emphasize the use of these preclinical setting to study tumor cell-immune cell interactions and to explore immunotherapeutic drug screens. We also investigate the application of these preclinical models to novel self-assembling drug development and discuss the challenges that need to be overcome to make possible a more widespread and rationalized use of PDX and PDO models. A careful consideration and evaluation must be given to the complexity of humanized PDX and PDO mice and their limitations. As a result, there will be a greater chance of achieving effective research results. In any case, we hope that the optimization of humanized PDX and PDO mouse models will make significant contributions to tumor immunotherapy and personalized medicine for improving the outcome of cancer patients in the future.

## 2 PDXs and PDOs models

### 2.1 PDXs

**Figure 1:**
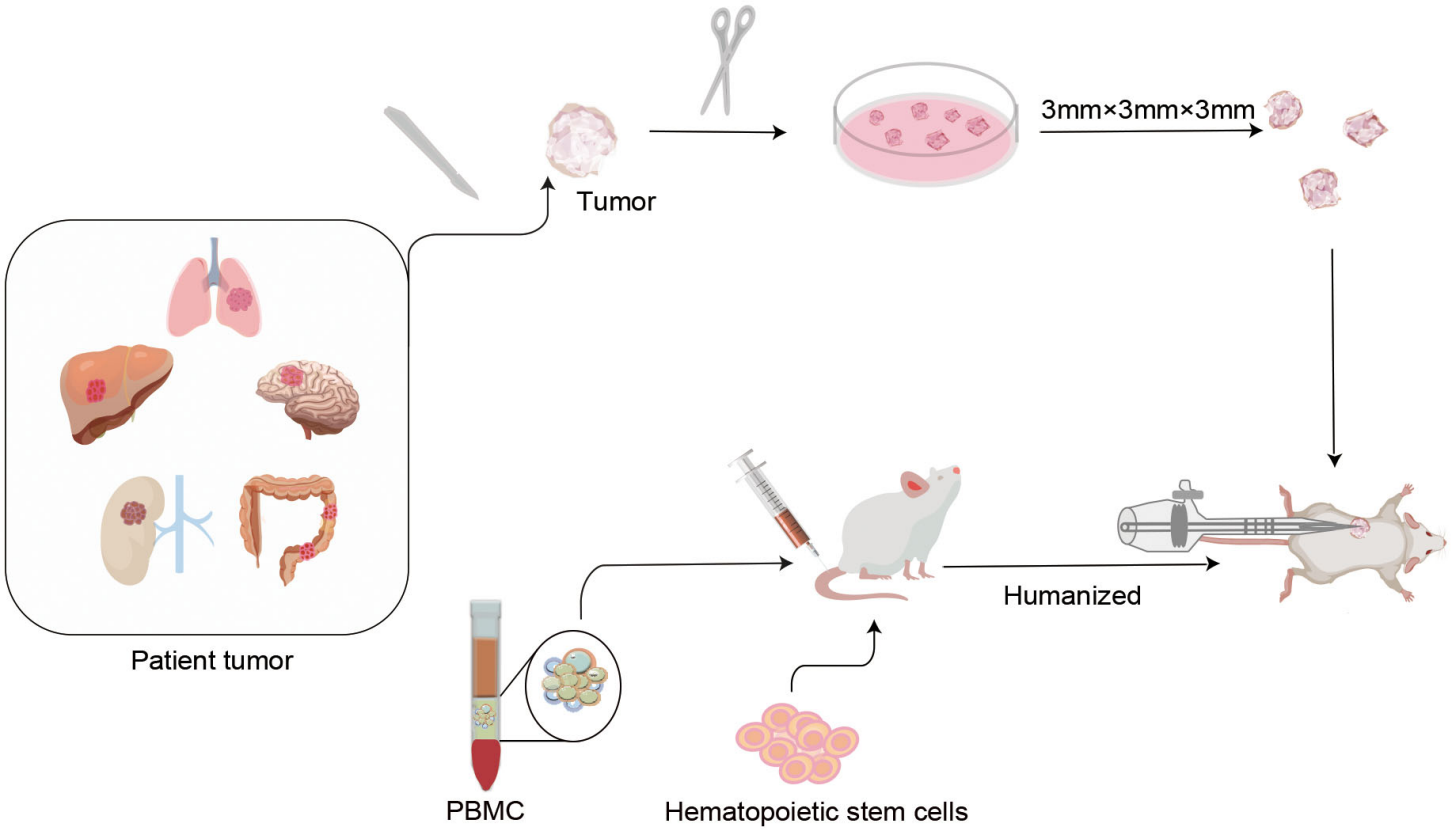
Protocols of humanized patient-derived tumor xenografts (PDX) model construction. In the first step, humanized mice were established by transplanting isolated human peripheral blood mononuclear cells (PBMC) or CD34+ human hematopoietic stem cells (HSC), etc. into severely combined immunodeficient mice. After the human immune system is successfully implanted, a novel humanized PDX model is established by inoculating patient-derived tumor tissues into humanized mice. These types of models not only mimic the phenotypic and molecular characteristics of the original tumor in the patient, but also reproduce the cross-talk between the tumor and the immune system. This is of critical significance for individualized drug marker screening and drug development for tumor immunotherapy.

Over the years, PDXs have been used to study several aspects of oncological diseases, especially for individualized drug development. It has been proposed that PDX models not only recapitulate key characteristics of human tumors with high fidelity, but also exhibit treatment responses that are concordant with human responses(28–30). In recent years, breakthroughs in tumor immunotherapy have placed increased demands on the development of appropriate preclinical assessment models to evaluate tumor immune responses. Therefore, humanized PDX models have been developed to evaluate the efficacy of immunotherapeutic approaches in cancer. The fundamentals of the humanized PDX model are as follow. In summary, pieces of solid tumors are obtained through surgery or biopsy procedures, and these samples are implanted into the dorsal region or the same organ of immunocompromised mice. To simulate a more realistic state of functional human immune system (HIS), human peripheral blood mononuclear cells (PBMC), CD34+ hematopoietic stem cells (HSC), or other immune cells can be transplanted into immunodeficient mice such as non-obese diabetes (NOD)- severe combined immune deficiency (SCID) gamma(NSG)mice. After human immune reconstruction, patient-derived tumor tissues can be transplanted to create a dual immunogenic model with similar heterogeneity and tumor immune microenvironment (TIME) as patients. This model can not only simulate the growth process of tumors in patients, but also simulate the interaction between a cancer cell and the HIS. The construction process of humanized PDX models is presented in **Figure 1**.

Humanized PDX models have provided a tremendous boost to the study of tumor pathogenesis and drug development. However, there are still limitations of humanized PDX models: 1) the time period required to build PDX models from patients is long and may take up to 6 months (or longer), 2) the high cost and low throughput, 3) lack of maturation of innate immune cells, coupled with insufficient ability to generate antigen-specific antibodies, 4) limited education of T cells in absence of murine thymus, 5)deficient HLA molecules, and 6) the difficulty in generating lymph node structures and germinal centers(31). These limitations have led to several ongoing efforts to develop novel humanized preclinical models and platforms to develop therapeutic strategies that enhance response to immunotherapy. In general, it is believed that the robustness of drug-screening data will increase when both human-derived immune reconstruction and data analysis become more standardized.

### 2.2 PDOs

**Figure 2:**
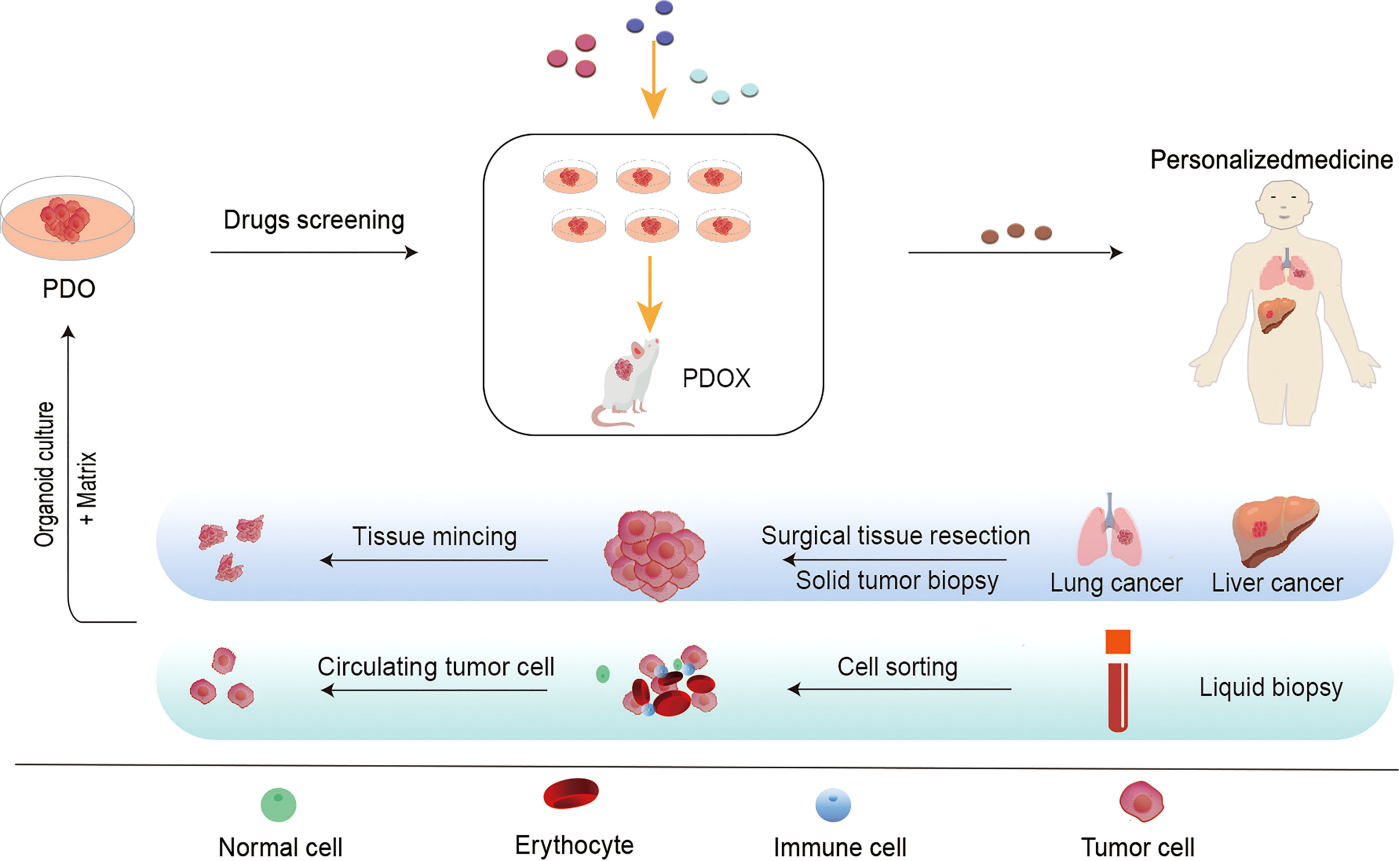
The flow chart of the establishment of patient-derived tumor organoid (PDO) models and the application of PDO in personalized treatment. It is commonly used to obtain primary tumor samples from surgical resections, puncture samples, or circulating tumor cells (CTCs) isolated from blood samples. Patient-derived cancer cells can be propagated *in vitro* on an enriched Matrigel matrix and cultured into three-dimensional tumor-like organs for *in vitro* and *in vivo* applications. These tumor models can be used for drug screening, gene analysis, immunotherapy, and other studies to accurately detect drug efficacy and toxicity. Furthermore, we are able to develop effective individualized treatment strategies for patients.

In 2009, Hans Clevers’ team successfully cultured mouse intestinal organoids that self-renew and maintain the villous structure of intestinal gland pits *in vitro*, bringing new starting for development of cancer therapeutic approaches (32). As an *in vitro* 3D organ, PDO can not only mimic the cell composition and structure in tumor growth, but also perform specific gene editing, which can satisfy complex tumor microenvironment research and potential drug screening. PDO and organoid-derived PDX(PDOX), as an emerging field, have attracted much attention since they can provide a cancer pre-clinical platform to recapitulate the patient’s tumor and promote translating novel treatments from bench to bedside (33–35). Over the past decades, several PDOs have been successfully cultured, including gastric tumors (36), breast tumors (37), bladder tumors (38), and ovarian tumors (39). The establishment and subsequent screening of PDO/PDOX can generally be completed in a shorter period than for PDX. The model construction process for PDO and PDOX is depicted in **Figure 2**.

Numerous studies in the past decades have demonstrated how using organoids enhances the accuracy of the drug screening system(37, 40). These PDOs have been widely employed in the research of anti-tumor drugs. There are many advantages associated with organoids, primarily in the realization of individualized precision medicine, the reduction of modeling time, high throughput screening, genomic screening, and drug screening(41, 42). Unfortunately, no single mouse model can capture every aspect of the parent tumor and immune landscape. Some major drawbacks should be considered. Organoids cannot perfectly replicate the microenvironment *in vivo*, they lack tumor blood vessels and immune cells, and the co-culture system with other cell types is not yet well established. There are difficulties in studying the role of other systems and organs within the body. A global standard for organoids establishment and quality control does not yet exist. Despite its limitations, it still provides an extremely valuable contribution to the research and development of cancer drugs(43). In any case, to maximize the potential for translational research, it is imperative to select the most suitable humanized mouse model (44).

## 3 PDX and PDO models in the discovery of traditional tumor immunotherapy drugs

### 3.1 Immune checkpoint inhibitors

Immune surveillance is a vital tool for inhibiting tumorigenesis and maintaining the body’s internal environment’s homeostasis. A tumor cell can self-modify or release factors that influence TIME, such as engaging immune checkpoint pathways, to evade immune surveillance. Several immune checkpoints have been identified, including PD-1 and its ligand (PD-L1), which regulate the activity of T cells and cancer growth (45). Since the development and clinical application of ICIs, cancer immunotherapy has significantly expanded our toolkit for fighting the disease. At present, ICIs primarily consist of antibodies against CTLA-4 (ipilimumab), PD-1 (nivolumab, pembrolizumab, cemiplimab) and PD-L1 (atezolizumab, durvalumab, avelumab) (15, 46, 47).

Researchers recently established a humanized mouse NPC-PDX model by engrafting nasopharyngeal carcinoma (NPC) biopsies in NSG mice. This model was used to investigate the anti-tumor efficacy of nivolumab and ipilimumab (48). A study published in 2019 evaluated the efficacy of nivolumab against colorectal cancer (CRC) in a hematopoietic humanized PDX mouse model (hu-CB-BRGS). It was observed that PD-1 blockade therapy induced the immune system to kill tumors in this mode of action (49). Kleinmanns et al. established a HIS-PDX model of ovarian carcinoma *in situ* of NSG mice, which were injected with CD34+HSC *via* the vein beforehand. They further investigated the change in immune cells by flow cytometry after the animal was treated with nivolumab. The results indicated that the overall response to monotherapy was modest, and the combination therapy might be more effective (50). Here, we summarized the conditions in which PDX models were used in the preclinical evaluation of immune checkpoint mAb drugs that are currently available, to better understand the utilization of the PDX model in drug development (**Table 1**).

**Table 1:** Patient-derived tumor xenografts (PDX) models in preclinical evaluation of immune checkpoint monoclonal antibody drugs.

Target	Name of drug	Type of tumor	Model and Strain	Reference
PD-1	Pembrolizumab and Nivolumab	TNBC	Hu-HSC-PDX (NSG mice)	(51)
PD-1	Pembrolizumab and Nivolumab	NSCLC	Hu-HSC-PDX (NSG mice)	(52)
PD-1	Pembrolizumab	NSCLC	Hu-PBMC-PDX (NSG mice)	(53)
PD-1	Pembrolizumab	HCC	Hu-HSC-PDX (NSG mice)	(54)
PD-1	Nivolumab	NSCLC	Hu-HSC-PDX (NSG mice)	(55)
PD-1	Nivolumab	MRCC	Hu-HSC-PDX (NSG mice)	(56)
PD-1	Nivolumab	CCA	Hu-PBMC-PDX (NSG mice)	(57)
PD-1	Pembrolizumab	Liposarcoma	Hu-HSC-PDX (NSG mice)	(58)
CTLA-4	Ipilimumab and Nivolumab	NPC	Hu-HSC-PDX (NSG mice)	(48)
PD-L1	Atezolizumab	NSCLC	Hu-PBL-PDX (NSG mice)	(59)
PD-L1	Durvalumab	NMIBC	Hu-PBMC-PDX (NOG mice)	(59)
PD-L1	Durvalumab	NMIBC	Hu-PBMC-PDX (NOG mice)	(60)

NSCLC, Non-small cell lung cancer; HCC, Hepatic cell carcinoma; TNBC, Triple-negative breast cancer; MRCC Metastatic renal cell carcinoma; CCA, Clear cell adenocarcinoma; Squamous cell carcinoma; NPC, Nasopharyngeal carcinoma; NMIBC, Non-muscle invasive bladder cancer; BC, Breast cancer; Hu, Human; HSC, Human stem cell; PBL, Peripheral blood lymphocyte.

To enable PDOs reproduce the TIME, researchers have developed a number of novel platforms to evaluate the efficacy of tumor immunotherapy in recent years. For example, researchers have constructed a complex air-liquid interface approach PDO from different cancer types, allowing *in vitro* preservation of the tumor epithelium and its stromal microenvironment, and even immunologically active CD8+ T cells, NK cells, etc. Using this model, it is possible to simulate the biological behavior and therapeutic response of tumors during anti-PD-1 therapy (61). By combining PDO and humanized mouse techniques, the investigators constructed a new model of spontaneous multi-organ metastasis from microsatellite instability-H CRC and also provided empirical evidence for a key role of B cells in generating site-dependent anti-tumor immunity after anti-CTLA-4 treatment(62). Researchers also demonstrated using a patient-derived organotypic tumor spheroids (PDOTS) and a matched PDO drug screening platform that inhibition of innate immune kinase TANK-binding kinase 1 coupled with PD-1 blockade was an effective strategy for overcoming tumor immunotherapy resistance (63). In addition, the investigators established a glioblastoma (GBO)-related organoid biobank for individualized therapeutic screening. This is a PDO model with significant clinical translational potential to simulate tumor response to CAR-T cell immunotherapy (64). These studies demonstrate that immuno-oncology studies can be successfully conducted using organoid models that may facilitate personalized immunotherapy testing. In order to better understand the advantages and disadvantages of the PDOs model. We also summarize the studies with the PDO model to evaluate ICIs drugs briefly (**Table 2**).

**Table 2:** Patient-derived organoids (PDOs) models in preclinical evaluation of immune checkpoint monoclonal antibody drugs.

Target	Name of drug	Organoids	Reference
PD-1	Nivolumab	Patient-derived gastric cancer organoids	(65)
PD-1	Pembrolizumab and Nivolumab	Patient-derived lung cancer organoids	(66)
PD-1	Nivolumab	Patient-derived chordoma organoids	(67)
PD-1	Nivolumab	Patient-derived renal cell carcinoma organoids	(68)
PD-1	Pembrolizumab and Cabozantinib	Patient-derived renal cell carcinoma clusters	(69)
PD-L1	Atezolizumab	Patient-derived renal cell carcinoma organoids	(70)

### 3.2 Direct immune modulators

Immunosuppressive cells (such as myeloid-derived suppressor cells and regulatory T cells) can release inhibitory cytokines in the TME to evade the immune system (71). Cytokines, such as interferon (INF)-alpha and interleukin (IL)-2, also play a crucial role in tumor immunotherapy. In 1986, the FDA approved INF-α as a cancer therapy drug for the treatment of leukemia. Currently, IFN-α and IL-2 have become the most widely used drugs in tumor immunotherapy strategies, however, several other cytokines are currently under clinical investigation (72, 73). Aside from cytokines, non-specific immune drugs also include target natural killer (NK) cells, macrophages, and immunomodulators. Pexidartinib, the first macrophage-targeting medicine approved by the FDA, is recommended for adult patients with symptomatic giant cell tumors of tenosynovitis because it restricts macrophage proliferation by blocking CSF cytokines (74). In a recent study on pexidartinib, researchers evaluated its impact on PDX and observed that pexidartinib can significantly inhibit osteosarcoma tumor growth (75). Immunotherapy with IL-2 and GM-CSF has significantly improved survival in children with high-risk neuroblastoma (76). Treatment failure and IL2-related toxicity, however, pose significant challenges to the treatment of one third of these patients. There has been evidence in recent clinical trials that NK cells hyperproliferate and acquire an activated phenotype in patients receiving recombinant human IL-15, resulting in NK cell expansion *in vivo* and tumor shrinkage in two patients. As a result, scholars validated the tumor suppressive effect of IL-15 on PDX models, and they demonstrated that the replacement of IL-2 with IL-15 was associated with significant tumor regression *in vivo*, supporting clinical trials of IL-15 for pediatric neuroblastoma (77). Additionally, related study has also demonstrated that IL-15 enhanced the anti-tumor activity of γδ T cells, and effectively suppressed tumor growth, and prolonged the survival of renal cancer-bearing PDX mice (78). Taking these results into consideration, it appears that cytokines might be able to have significant clinical implications in the future.

### 3.3 CAR-T/NK

CAR- T cell therapy, as a novel approach in anticancer therapy, in which T cells are retargeted against the tumor cell following the engineered expression of CARs (79). Currently, two CAR-T cell products have been used for the treatment of lymphoblastic leukemia and lymphoma (80). Besides, it has been reported that CAR-T cells engineered to simultaneously produce interleukin (IL)-7 and chemokine (C–C motif) ligand 19 (CCL19) were effective against solid tumors in pancreatic cancer (PC) PDX model (81). Additionally, other researchers have also verified CAR-T cells anti-tumor immunotherapy effects on triple-negative breast cancer(TNBC) (82). Other studies have found that in a patient with late-stage HCC, anti-GPC3 IL-7/CCL19 CAR-T therapy resulted in complete tumor disappearance 30 days post-intra-tumor injection. And in a patient with advanced PC, anti-MSLN-IL-7/CCL19 CAR-T cellular therapy resulted in almost complete tumor disappearance 240 days post-intravenous infusion (83). Both preclinical and clinical studies suggest that novel CAR-T cells have significant potential for the treatment of solid tumors.

In 2010, Zhao Y et al., reported that they developed a PDX model to evaluate CAR-T therapy (84). Jiang Z et al., reported that CAR T cells demonstrated a positive therapeutic effect on liver cancer in a PDX mouse model. They concluded that the growth of the tumor in the PDX model could be inhibited following CAR-T cells therapy (85). The investigators developed a highly specific SynNotch-CAR-T cells, which was validated using the PDX model to target gliomas and exert anti-tumor effects with reduced off-target toxicity (86).The emergence of adaptive therapy has stimulated the development of new CAR-NK cells therapy techniques (20). In 2021, Cao B and his team developed mesothelin (MSLN)-CAR NK cells, which were evaluated using PDX (NSG mice). According to the findings, these cells demonstrated strong anti-tumor properties and offer a promising treatment for gastric cancer (87). Although CAR-NK cells have obvious advantages in tumor therapy, the short life cycle of NK cells *in vivo* and the immunosuppression of the TME limit the clinical transformation of CAR-NK cells.

Ding S et al., generated thousands of micro-organ spheres from patient tissues and assessed tumor drug response (88). The establishment of an organoid biobank, as mentioned earlier, is a valuable platform for evaluating tumor treatment strategies such as CAR-T cell therapy (89). In addition, combining organoid and 3D imaging technologies, the investigators have provided a platform to reveal the mode of action of cellular anti-cancer immunotherapies in a patient-specific manner and apply them to develop multiple engineered T cell products(90). PDOs are ideal for short drug screening cycles and convenient sampling of the model, which can be achieved through several methods, including surgery, biopsy, urine, and lung lavage fluid (88, 91). The development of PDOs will greatly shorten preclinical study time and facilitate drug development.

### 3.4 Oncolytic viruses

More than a century ago, a phenomenon was observed in clinical therapy, that is some patients with cancer experience the cancer regression if they were infected with certain viruses (92). Based on this case, OVs therapy was further developed to advance cancer biological therapy. OVs possess excellent safety in clinical trials, which greatly promotes their research and development. A novel OV (OAd-MUC16-BiTE) with better anti-tumor characteristics was developed for treating ovarian cancer in PDX mice models (93). Other study evaluated the anticancer efficacy of VG161, a herpes virus type 1 (HSV-1), in HLA-matched CD34+ humanized PDX model. It was found that VG161 significantly inhibited tumor growth and would realize enhancement of OV-induced antitumor immunity for long-term maintenance treatment (94). In research by Quinn CH et al., oncolytic herpes simplex viruses (oHSVs) were demonstrated to be effective in treating high-risk neuroblastoma in PDX mice (NOD-SCID) (95). OVs therapy has the advantages of excellent replication efficiency, a potent killing effect, fewer adverse reactions, and inexpensive cost, making it one of the most promising tumor immunotherapy methods in the future (21, 33). In addition, exploring the anticancer activity of OVs based on pancreatic PDOs proved to be an effective predictive tool (96). However, the delivery of OVs was by intertumoral injection, which limited its clinical use. Therefore, how to deliver these OVs to the tumor tissue more effectively and how to improve the potential of these viruses to disseminate within the tumor tissue site may be the future focus of this therapy.

## 4 PDXs and PDOs in the discovery of self-assembled drugs for tumor immunotherapy

Tumor immunotherapy has changed the treatment of advanced tumors, however, the proportion of patients responding to immunotherapies remains low. In recent years, supramolecular chemistry and self-assembled systems have been extensively investigated in the field of cancer therapy and hold great promise for improving immunotherapeutic outcomes in tumor patients (97, 98). Unlike conventional cancer immunotherapy, rationally designed nano-self-assembled drugs can trigger specific tumoricidal effects, thereby improving infiltration of TIME such as killer immune T lymphocytes, optimizing antigen presentation, and inducing durable immune responses (23). The development of nanotechnology provides an opportunity for enhancing the effectiveness and reducing the toxicity of traditional drugs, and a series of nanomaterials were used in the preclinical study of cancer (24). In conclusion, self-assembled drugs have a broad potential for application in tumor immunotherapy, especially in refractory and recurrent cancers.

The self-assembled peptides can respond to various environmental conditions, such as pH, temperature, and molecular interactions, while also possessing high biocompatibility and drug loading capabilities(99, 100). According to current research, self-assembly peptides can be classified into two main categories for tumor immunotherapy research: 1) Self-assembly into nanodrugs using their drug loading capacity, delivering molecules such as peptides or siRNA, inhibiting specific proteins or genes in tumor cells to enhance tumor immunotherapy response. 2) Using peptide self-assembly to simulate tumor antigens as tumor vaccines to stimulate the body to produce anti-tumor antibodies(101, 102). In short, self-assembling peptide drugs may improve the treatment of tumors immune therapy significantly in future.

Self-assembled nanomedicines have received significant attention due to their excellent biocompatibility, high modification versatility and ease of synthesis, controllable and adaptive nanostructures(103). Recently, a study pointed out that through targeted inhibition of MDM2, p53 can be activated, the tumor immune microenvironment can be reprogramed, and immunotherapy resistance can be overcome (104). Researchers created TPA, a combined targeted peptide that inhibited the PD-1/PD-L1 axis, activated p53, and showed tumor killing and immunotherapeutic sensitization effects on a humanized PBMC-engrafted PDX model. There is now a potential pathway for the development of self-assembled peptide drugs for cancer therapy (105). For tumor targeting, the researchers synthesized size-tunable nanostructures with a spherical morphology by combining partially reductive HSA with hydrophobic Fluvastatin, known as AB-Flu. According to the study, these nanodrugs effectively enhanced the potency of Anti-PD1 antibodies against colon cancer in a humanized CRC-PDX mouse model while maintaining acceptable levels of safety (100). Generally, self-assembled drugs have unique anti-tumor effects and are low in toxicity. Through blocking the supply of tumor nutrients, improving drug targeting, and even recruiting multiple immune cells, they can achieve tumor therapy. Therefore, development and research into self-assembled drugs is warranted. Anti-tumor potential of self-assembled drugs creates new hope for tumor treatment, and the PDX and PDO models facilitate clinical transformation as well.

## 5 Perspective and conclusion

The rapid development of immunotherapy drugs brings hope to clinical patients with cancer (100, 106–108). However, the preclinical evaluation of drugs still restricts the development of drug research. Although the drug evaluation system has advanced from a 2D cell line evaluation system to PDX/PDO system and even developed a PDX model with human immune cells to more accurately simulate the immune environment *in vivo* (109, 110), there are still limitations. The establishing cycle of PDX/humanized PDX is long, the technology is challenging, and it cannot completely simulate the TME. Although the modeling cycle of PDOs is short, but it is still necessary to investigate whether the medium possesses antigenicity because the composition of the medium is unknown. In addition, despite the extensive genetic heterogeneity of tumors *in vivo*, it is unknown whether tumor organoids can capture the entire range of heterogeneity that originates from the primary tumor (111). Despite the complexity of cancer, there are still several unresolved issues, including those related to its pathogenesis, mechanism of metastasis, patient response to treatment, or mechanism of drug resistance. Further studies are required to constantly improve the simulation of TME to create PDX/PDO that is more similar to the primary tumor characteristics, to better serve drug development.

## Author contributions

WZ wrote the manuscript. XZ reviewed and edited the manuscript. All authors contributed to the article and approved the submitted version.
